# PTG Depletion Removes Lafora Bodies and Rescues the Fatal Epilepsy of Lafora Disease

**DOI:** 10.1371/journal.pgen.1002037

**Published:** 2011-04-28

**Authors:** Julie Turnbull, Anna A. DePaoli-Roach, Xiaochu Zhao, Miguel A. Cortez, Nela Pencea, Erica Tiberia, Mark Piliguian, Peter J. Roach, Peixiang Wang, Cameron A. Ackerley, Berge A. Minassian

**Affiliations:** 1 Program in Genetics and Genome Biology, The Hospital for Sick Children, Toronto, Canada; 2 Department of Molecular Genetics, University of Toronto, Toronto, Canada; 3 Department of Biochemistry and Molecular Biology, Indiana University, Indianapolis, Indiana, United States of America; 4 Division of Neurology, Department of Paediatrics, The Hospital for Sick Children, Toronto, Canada; 5 Department of Pathology and Laboratory Medicine, The Hospital for Sick Children, Toronto, Canada; The Jackson Laboratory, United States of America

## Abstract

Lafora disease is the most common teenage-onset neurodegenerative disease, the main teenage-onset form of progressive myoclonus epilepsy (PME), and one of the severest epilepsies. Pathologically, a starch-like compound, polyglucosan, accumulates in neuronal cell bodies and overtakes neuronal small processes, mainly dendrites. Polyglucosan formation is catalyzed by glycogen synthase, which is activated through dephosphorylation by glycogen-associated protein phosphatase-1 (PP1). Here we remove PTG, one of the proteins that target PP1 to glycogen, from mice with Lafora disease. This results in near-complete disappearance of polyglucosans and in resolution of neurodegeneration and myoclonic epilepsy. This work discloses an entryway to treating this fatal epilepsy and potentially other glycogen storage diseases.

## Author Summary

Lafora disease (LD) is a fatal epilepsy that afflicts previously normal teenagers. It is caused by mutations in the *EPM2A* or *EPM2B* genes encoding the laforin carbohydrate-binding phosphatase and the malin E3 ubiquitin ligase. LD is the most common neurodegenerative epilepsy of adolescents. Affected children suffer an ordeal lasting 10 years, consisting of escalating seizures, constant body jerking, particularly frightening epileptic visual hallucinations, and later on dementia. They die of massive convulsion. Brain biopsies reveal accumulation of a starch-like compound, polyglucosan, overtaking dendrites and likely causing the disease, and neurodegeneration. Glycogen synthase (GS), the enzyme that forms normal glycogen, is also responsible for synthesizing these polyglucosans. We reasoned that reducing GS activity might prevent polyglucosan formation. Mice deficient of *Epm2a* replicate LD and are a standard model. Members of our group generated mice deficient of PTG, a protein involved in activating GS. By breeding LD mice with PTG-lacking mice, we generated LD mice lacking the GS-activating effect of PTG. This resulted in a cure. The double knockout mice have almost no polyglucosan, no neurodegeneration, and no seizures. Our work opens an avenue of treatment for this fatal epilepsy, which may also be applicable to other glycogen storage diseases.

## Introduction

Lafora disease (LD) is caused by recessively inherited mutations in the *EPM2A* or *EPM2B* genes, encoding laforin (a carbohydrate binding phosphatase) and malin (an E3 ubiquitin ligase) [1], [2]. The disease begins around age 15 with myoclonus (jerk-like seizures) and generalized convulsive seizures, which initially respond to medications. Over the next five years seizures become intractable and the myoclonus near-constant, and epileptic hallucinations with highly frightening content appear. Extremely frequent myoclonic seizures (repetitive jerks) and epileptic absence attacks permeate consciousness and prevent formulation of complete thoughts. Dementia and a vegetative state in constant myoclonus follow. Death occurs around age 25 in status epilepticus. Pathology consists of the progressive formation of polyglucosans, which are insoluble glucose polysaccharides that precipitate and aggregate into concretized masses called Lafora bodies (LB), and in neurodegeneration. LB form in neuronal perikarya (i.e. in the cell body near the nucleus) and in neuronal short processes (mostly dendrites). LB in the neuronal processes are much smaller but they massively outnumber LB in the perikarya. Extraneurally, LB also form in heart, liver, and skeletal muscle, but cause no symptoms in these organs [3]–[6].

A normal glycogen molecule contains up to 55,000 glucose units, yet remains soluble because its glucose chains are short (13 units), each chain is a branch of another, and the whole molecule is a sphere, the surface of which is composed of the hydrophilic ends of chains [7]. This unique organization allows mammalian cells to store large amounts of carbohydrate energy in a soluble rapidly accessible form. Without branching, glucose polymers 13 units or longer are poorly soluble and tend to precipitate and crystallize [8]. Polyglucosans are malformed glycogen molecules. They have very long chains, insufficient branches, and a resultant lack of spherical organization. They are more similar to plant amylopectin or starch than to glycogen, and like these plant carbohydrates they are insoluble, precipitate, and accumulate [3], [5], [9].

Glycogen is synthesized through coordinated actions of glycogen synthase (GS) and glycogen branching enzyme, the former responsible for chain elongation, the latter for chain branching. Glycogen is digested by glycogen phosphorylase (GP) and glycogen debranching enzyme. PTG (protein targeting to glycogen) is an indirect activator of GS and an indirect inhibitor of both GP and glycogen phosphorylase kinase (GPK), the enzyme that activates GP. PTG performs this reciprocal activation of synthesis and inhibition of breakdown by binding the pleiotropic phosphatase PP1 through its C-terminus, binding glycogen, and through a common region in its N-terminus (amino acid sequence WDNNE) binding GS, GP, or GPK, thus targeting PP1 to each of the three enzymes. PP1 dephosphorylates each of the three enzymes, activating GS and inhibiting GP and GPK [10], [11].

There are two main hypotheses of polyglucosan formation, the first based on evidence from cell models that laforin interacts with malin and with PTG, and that the laforin-malin complex downregulates GS through malin-mediated ubiquitination and degradation of PTG. In this hypothesis, absence of laforin or malin would increase PTG, which would over-activate GS, leading to excessive extension of glycogen chains and conversion of glycogen to polyglucosan [12]–[14]. Although results from animal models have yet to confirm this idea [15]–[17], there is indeed a body of work implicating PTG.

The second hypothesis is based on the observation that laforin dephosphorylates glycogen and that in LD there is progressive hyperphosphorylation of glycogen, causing it to unfold and precipitate. GS remains bound to the precipitating glycogen, but glycogen branching enzyme, the enzyme responsible for branching, even under normal condition does not associate tightly [16]–[19]. In this hypothesis, elongation by GS of the chains of the precipitated glycogen, with no branching, would convert glycogen to polyglucosan. Both hypotheses predict that inhibiting GS would prevent polyglucosan formation, and if LB are causative of the PME, this might ameliorate or cure the epilepsy. One way to inhibit GS would be to interfere with its activation by PTG. In the present work we genetically remove PTG from mice with LD. We obtain dramatic reduction in LB, and resolution of neurodegeneration and the PME. This work has direct implications for therapeutic intervention in this fatal disease.

## Results

### Generation of laforin-deficient mice lacking PTG

We initially considered removing the muscle/brain isoform of GS (GYS1) from LD mice by breeding GYS1-deficient mice with laforin-deficient mice. However, this is impractical because in 90% of cases GYS1-deficient mice cannot survive birth (although the 10% that do are subsequently healthy with normal lifespan and exercise tolerance) [20], [21]. Recently, DePaoli-Roach generated a mouse line deficient of PTG. In contrast to an earlier report that disruption of the PTG gene was embryonic lethal [22], the present mice are healthy and have normal lifespan [23]. Their glycogen is reduced by 30% in skeletal muscle and by 70% in brain.

Laforin-deficient mice (LKO) have been extensively characterized and exhibit LB formation, neurodegeneration, and PME [24]. The PME is not as severe as in humans. The mice develop progressively worsening myoclonus, but convulsive seizures are not seen [24]. Unlike human patients and despite the neurodegenerative changes and progressive myoclonus LKO mice do not have a shortened lifespan (unpublished observation). Metabolically, LKO mice have progressively increasing accumulation of glycogen in tissues, reaching approximately fivefold normal in brain and threefold in skeletal muscle by age nine to 12 months [16]. To remove PTG from the laforin-deficient mice, we bred LKO mice with PTG knockout mice and interbred their litters to produce PTG/laforin double knockout (DKO) animals. DKO mice are born at Mendelian frequency, have normal skin, body habitus and growth, exhibit no obvious behavioral abnormalities, and appear to have normal lifespan, our oldest presently healthy at 18 months of age.

### Laforin-deficient mice lacking PTG have greatly decreased Lafora bodies and normal glycogen levels

**Figure 1:**
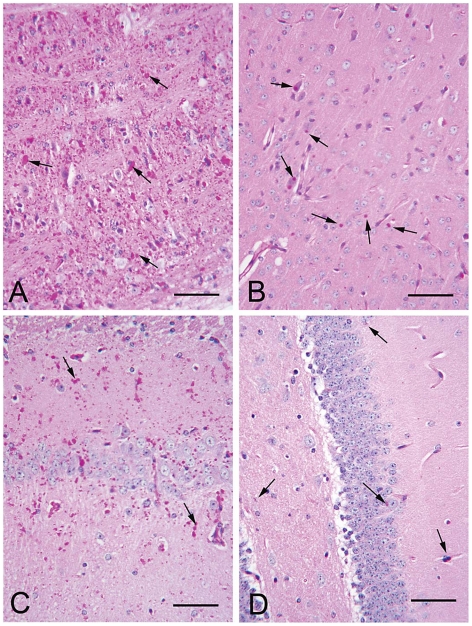
LB in brain of 12 month-old LKO and DKO mice. (a–b) Frontal cortex and hippocampus respectively from a LKO mouse stained with PAS-D. Note abundant LB within the neuropil and in the perikarya of numerous neurons. (c–d) Comparable regions from a DKO mouse. Arrows, examples of LB. All bars, 50 µm.

**Figure 2:**
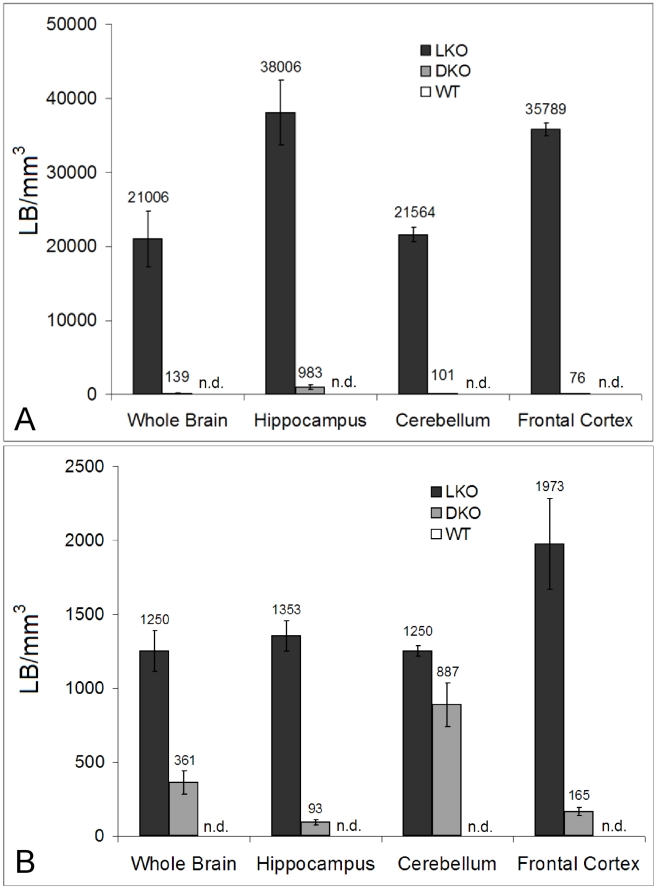
LB numbers in brain. (a) Morphometric analysis of granular LB in whole brain and different brain regions. Granular is the histochemical description of the small LB in the neuropil, which by electron microscopy are shown to be in neuronal processes, mainly dendrites. Statistics: p<0.001 in all regions between LKO and DKO (ANOVA); n = 4 per genotype. (b) Morphometric analysis of perikaryal LB. Statistics: p<0.001 between LKO and DKO, except in cerebellum, where the difference is not significant (ANOVA); n = 4 per genotype.

**Figure 3:**
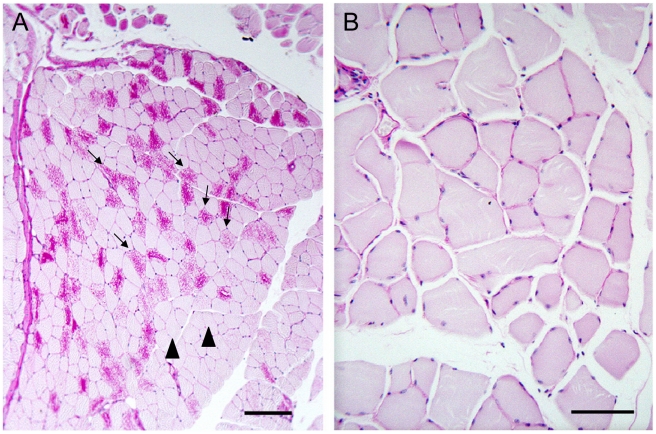
LB in skeletal muscle. (a) Muscle from a LKO mouse stained with PAS-D. Note presence of numerous LB in many fibers; bar, 100 µm; arrows, LB-replete myofibers; arrowheads, myofibers not containing LB. (b) Comparable field from a DKO mouse; bar, 50 µm. Higher magnification chosen for the DKO example to illustrate lack of even small LB.

**Figure 4:**
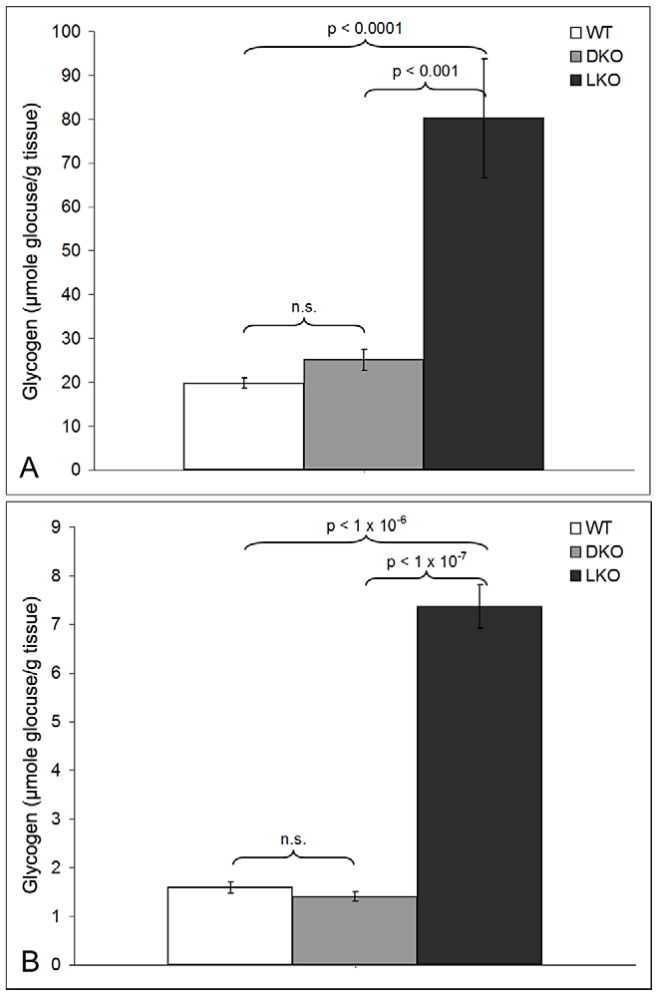
Glycogen levels in skeletal muscle and brain in 12-month-old wt, LKO, and DKO mice (µmol glucose/gm tissue). Skeletal muscle (a) and brain (b).

As mentioned, nine to 12 month-old LKO mice have vast amounts of LB in brain and other organs, and neurodegeneration [24]. We studied brain and skeletal muscle from LKO and DKO mice and their wild-type (wt) littermates at 12 months and found massive reduction in LB in DKO mice (Figure 1 and Figure 2). In hippocampus, frontal cortex and cerebellum, the numbers of LB in neuronal processes in DKO were respectively 3%, 0.1%, and 0.5% of those in LKO animals. The numbers of perikaryal LB were diminished to 10% in hippocampus and 5% in frontal cortex. In cerebellum, perikaryal LB were not significantly reduced in number, although they were much smaller in size. In skeletal muscle, LB had completely disappeared, compared to their very large quantities in LKO animals (Figure 3). Wt animals, as expected, had no LB in either tissue. To determine whether the reductions in LB correlated with reductions in glycogen content, we measured total glycogen in whole brain and skeletal muscle and found that the increased glycogen content of LKO mice had normalized to wt levels in DKO animals (Figure 4).

### Absence of PTG rescues neurodegeneration in laforin-deficient mice

**Figure 5:**
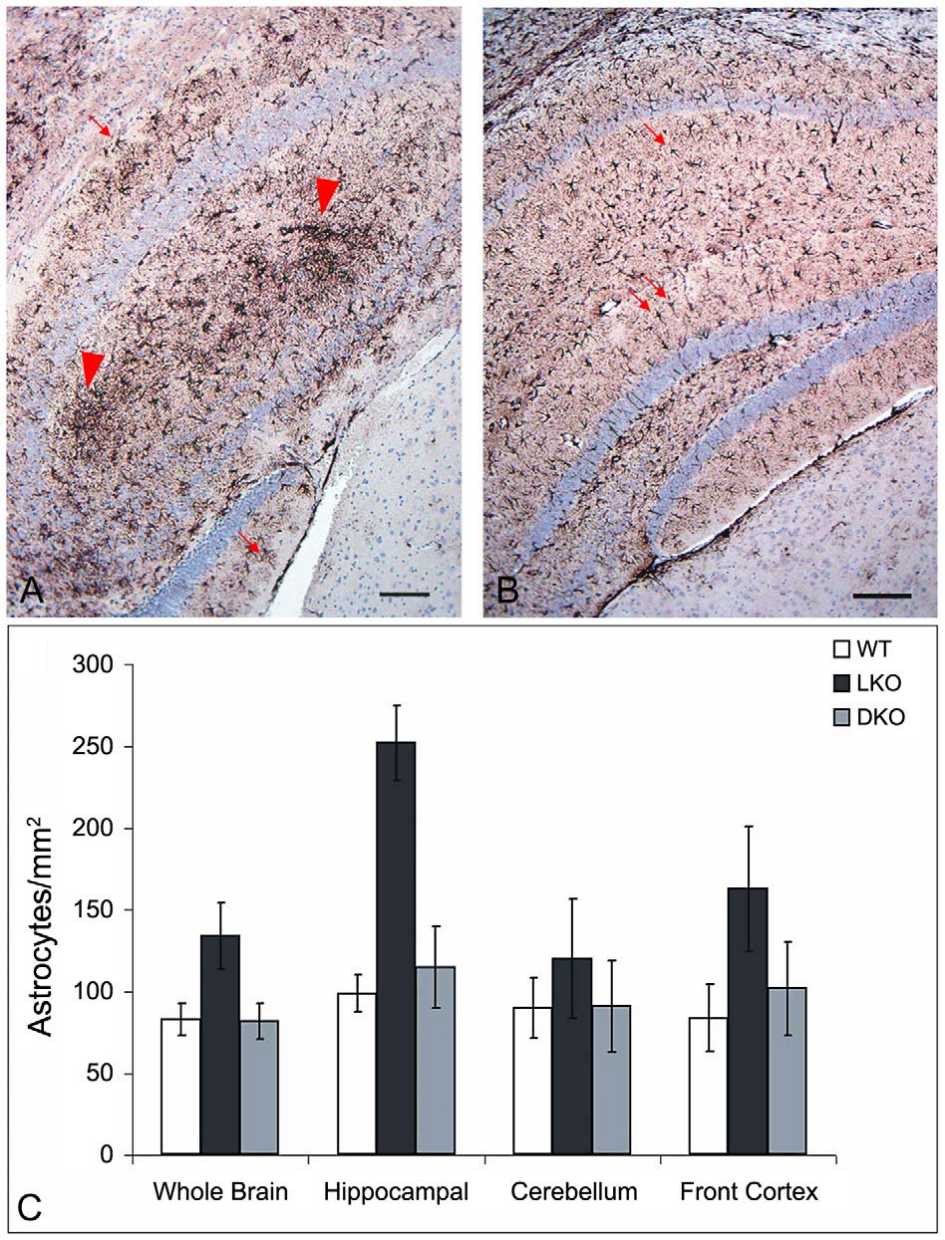
Gliosis in LKO mice. (a) Hippocampus of a LKO mouse stained with GFAP. Note the large numbers of GFAP-positive astrocytes. (b) Comparable region from a DKO mouse. Bars, 100 µm. Arrows, astrocytes; arrowheads, gliosis. (c) Counts of GFAP-positive astrocytes. For significance, whole brain p<0.02; hippocampus p<0.001; cerebellum, not significant; frontal cortex, p<0.002 (ANOVA); n = 4–7 per genotype.

**Figure 6:**
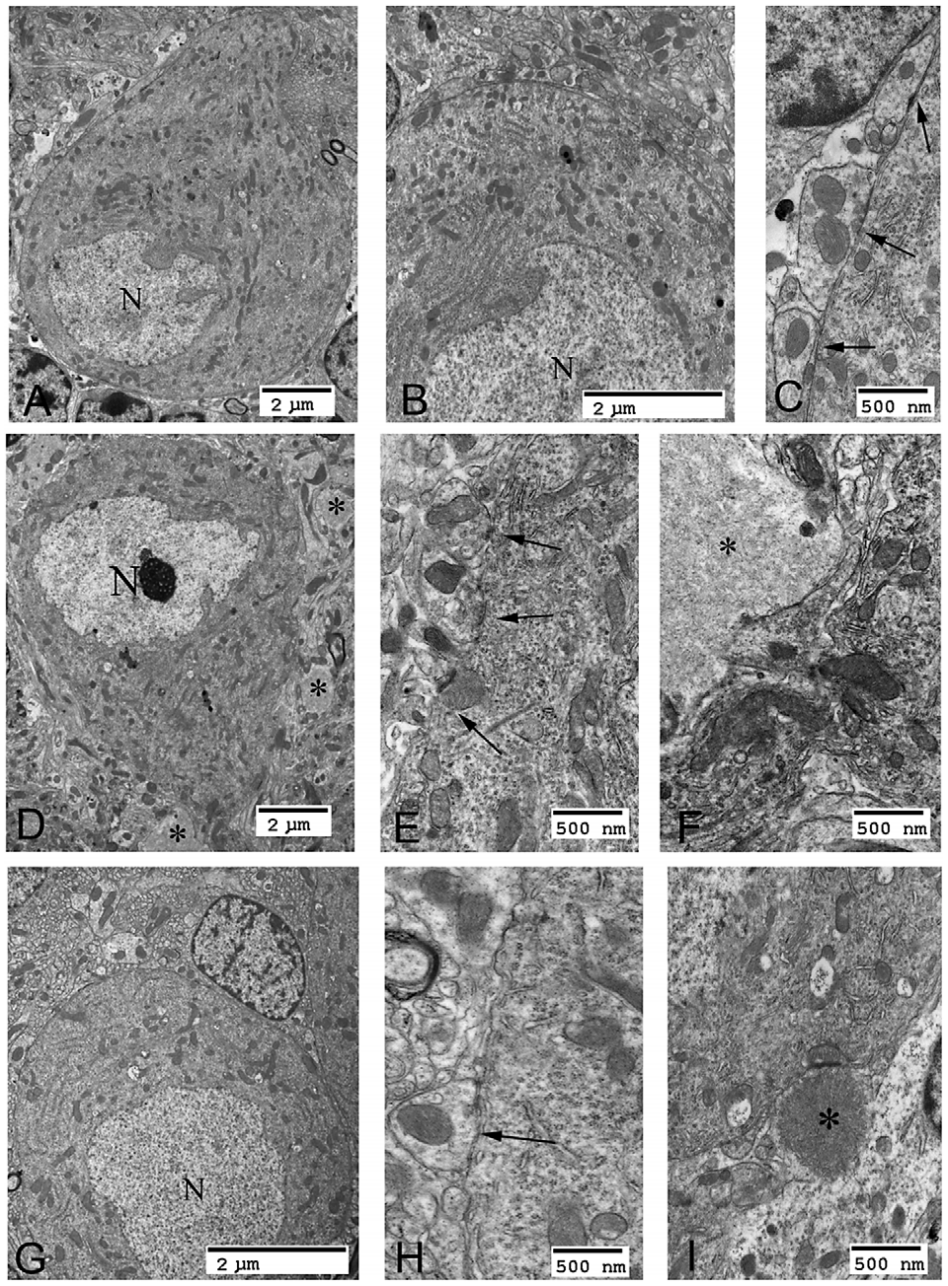
Neurodegeneration in LKO mice. (a–c) Cerebellar Purkinje cells from a wt mouse. Note the smooth appearance of the plasma membrane and the absence of any voids between the cell and the surrounding neuronal processes; N, nucleus; arrows, synapses. (d) Purkinje cell from a LKO mouse. Numerous LBs are seen surrounding the cell (asterisks). (e) Higher power of an LKO Purkinje cell; arrows indicate wrinkling and retraction of plasma membrane. (f) A large LB (asterisk) in close proximity to a degenerating LKO Purkinje cell. (g) A typical DKO Purkinje cell. Note its full normal appearance and smooth plasma membrane. Part of a second normal Purkinje cell is seen to the right of the panel. (h) Higher power of plasma membrane from a DKO Purkinje cell. Note the relative linear appearance of the membrane and the attached synapses (arrows) typical of a normal cell. (i) One of few LB (asterisk) near Purkinje cells detected by EM in DKO.

Lost neurons are replaced by astrocytes. We assessed neuronal loss in DKO, LKO and wt animals at 12 months first by measuring gliosis, which we quantified by morphometric counts of glial fibrillary acidic protein (GFAP)-positive cells. In cerebellum, there were no differences between the genotypes. In hippocampus and frontal cortex, however, DKO mice had half the number of astrocytes as LKO animals, and the same number as wt, i.e., they have no measurable gliosis (Figure 5). We next assessed neurodegeneration directly. In their original study of neuropathology in LKO mice, Ganesh and colleagues noted absence of apoptosis and necrosis. Using electron microscopy (EM), they documented an unusual form of somatic degeneration characterized chiefly by shrinkage and retraction of plasma and nuclear membranes and darkening of the cytoplasm [24]. We performed EM studies in the present set of LKO, DKO and wt mice. Figure 6A–6C show representative wt cerebellar Purkinje neurons with characteristic full nuclei and cytoplasms and taut and circular plasma membranes. Numerous axon terminals are seen directly apposed to the membranes forming normal synapses lined one next to the other around the circumferences of the cells. Figure 6D–6F show typical LKO Purkinje cells. Nucleus and cytoplasm are shrunken. The plasma membrane is wrinkled and retracted with appearance of indistinct spaces between it and the axon boutons that would normally associate with it, effectively resulting in loss of synaptic contacts. Numerous LB in neuronal processes are present. Figure 6G–6I show representative DKO Purkinje cells. The cells are essentially back to normal with full nuclei and cytoplasms, circular plasma membranes, and generally a full complement of synapses around the cell body. However, the correction while near-perfect is not completely perfect. The plasma membrane is not quite as taut as in wt, and there are rare instances of synaptic contact loss.

### Absence of PTG rescues the myoclonus of laforin-deficient mice

**Figure 7:**
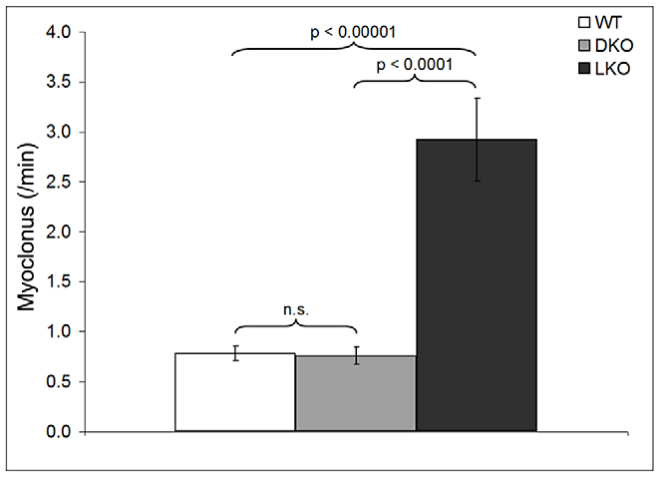
Myoclonus per minute in wt, DKO, and LKO mice. n = nine to 12 mice per genotype.

Myoclonus is a single jerk of the body or of a body part. Mice, like humans, exhibit a certain amount of physiologic myoclonus, such as hypnagogic myoclonus [25], [26]. In LD patients, myoclonus is extremely frequent and in later stages near-constant and debilitating [4], [5], [25]. We counted myoclonus in 12 month-old wt, LKO, and DKO animals, blind to genotype. Myoclonus was defined as sudden rapid jerks of the head or of the dorsum of the animal. In the latter, the split-second myoclonus causes retropulsion of the animal, closely resembling the myoclonus we documented previously in canine LD [27]. LKO mice have fourfold increased myoclonus over wt. DKO were the same as wt (Figure 7). In their original description Ganesh and colleagues reported that in addition to myoclonus 80% of nine to 12 month-old LKO animals also exhibit myoclonic seizures (polymyoclonus), consisting of rapid repetitive head and body jerks lasting few seconds and associated with epileptic discharges on electrocorticography [24]. We observed polymyoclonus in 80% of the present 12 month-old LKO mice, in no wt mice, and in no DKO mice.

## Discussion

In this study we show for the first time that removal of PTG in an animal model of LD reduces LB formation, and eliminates neuronal loss and the myoclonic epilepsy. PTG is not the only protein that targets PP1 to glycogen and glycogen metabolizing enzymes. Others include R6, which like PTG is ubiquitously expressed, R_GL_/G_M_ specific to striated muscle, and G_L_ found in rodent liver [7], [28]. It is therefore not surprising that skeletal muscle and brain of PTG-deficient mice still make glycogen, 70% and 30% of normal respectively [23]. What is surprising is the complete absence of LB in skeletal muscle in DKO. It would have been expected that if there is 70% glycogen synthesis in the absence of PTG, there would be 70% LB formation in the absence of laforin and PTG. Possibly, LB formation requires a threshold amount of glycogen. Alternatively, the laforin-malin complex in skeletal muscle acts specifically through PTG. On the other hand, if PTG is the preferred mediator of laforin-malin, it is surprising that its elimination from brain results in incomplete disappearance of LB, despite deeper glycogen reduction in brain in PTG deficient mice than in muscle. Much work ahead is needed to resolve these paradoxes.

The cause of neurodegeneration in LD has received much attention in recent years. Presence of up to 28% protein in some LB [9], [29], and signs of neurodegeneration in LKO mice at two months of age when LB are still small [24], led to considerations as to whether the neurodegeneration is related not to polyglucosans but to protein aggregation, similar to Alzheimer's and other neurodegenerative diseases [24], [30]–[32]. In the present study, correction of the neurodegeneration through interference in glycogen metabolism suggests that the neurodegeneration is connected to the disturbance in glycogen metabolism. This is consistent with recent observations that neurons, unlike other cell types, are highly vulnerable to increases in glycogen and polyglucosan content, with upregulation of GS leading to cell death [13]. Presence of small LB in two month-old LKO mice indicates that polyglucosans were already formed and accumulating by that time, likely triggering cell death, even as they had not yet formed large LB. Proteins in LB could be glycogen-metabolizing and other proteins trapped amidst aggregating polyglucosans.

Recently, it was reported that laforin enhances macroautophagy and that macroautophagy is dysfunctional in LD [33], indicating that laforin might function not only to prevent polyglucosan formation but also in clearing polyglucosans when they do form. Our results show that preventing polyglucosan formation obviates other laforin functions and suffices to prevent LD in mouse.

A major question in LD is why this particular neurodegenerative disease exhibits extremely severe epilepsy. Polyglucosans and LB occur in one other neurological disease, Adult Polyglucosan Body Disease (APBD), caused by mutations in the glycogen branching enzyme gene [34]. APBD LB differ from LD LB in one respect. For reasons unknown, they form exclusively in axons, especially long axons traveling to and from peripheral structures (skin, muscle, etc.) and the central nervous system. Affected patients suffer from motor neuron disease, may have mild dementia, but have no epilepsy [34], [35]. LD LB, on the other hand, are not seen in long tract axons, but instead almost completely replace the cytoplasm of vast numbers of small neuronal processes, mainly dendrites [3], [5], [6]. One possibility for the intractable epilepsy in LD is the progressive disturbance of dendritic function, the chief determinant of a neuron's excitability state. Near-complete disappearance of dendritic LB in the present study may account for the correction of the PME in our DKO mice.

In this paper, we correct the pathology and eliminate the PME of LD through genetic depletion of one of the proteins that targets the PP1 phosphatase to glycogen and the glycogen metabolizing enzymes. The effect on glycogen is partial, i.e. glycogen is not altogether eliminated, only reduced, the reduction returning the elevated glycogen levels of LD to normal wt levels, correcting the cardinal features of the disease, and causing no apparent harm to the mice. The crystal structures of PP1 [36], GS [37], [38], GP [39], [40], and GPK [41] are known, as is the PTG interaction domain with GS, GP and GPK [10], [12]. Identification of inhibitors of this interaction through rational design or large-scale small molecule screens could result in a treatment for this fatal epilepsy.

In addition to LD, accumulation of normal or abnormal glycogen is a cause of disease in several glycogen storage diseases including APBD and its severe fatal infantile form Andersen's disease [42], and the common and debilitating glycogenosis Pompe disease (acid maltase deficiency) [43]. Our results in LD suggest that removal of PTG could also improve these diseases. In fact, GS itself was recently removed from a Pompe mouse model resulting in a cure of the disease in that model [44]. While complete elimination of GS in humans cannot be contemplated as this causes significant pathology including sudden cardiac death [45], the Pompe study and our present work suggest that classes of medications that partially reduce GS or that partially reduce GS and activate GP, e.g. through interference in the PTG – GS/GP/GPK interaction, could have therapeutic benefit in multiple glycogenoses.

## Methods

### Ethics statement

All animal procedures were approved by the Toronto Centre for Phenogenomics Animal Care Committee.

### Immunohistochemical staining

Laforin-deficient mice were a gift of Dr. AV Delgado-Escueta and S Ganesh. Mice were sacrificed by cervical dislocation and tissues immediately fixed in 10% formalin. Periodic acid-Schiff-diastase (PAS-D) staining was as previously described [17]. PAS stains normal glycogen and polyglucosans. The short treatment with diastase (amylase) digests glycogen but not polyglucosans. Diastase resistant PAS stained structures are LB. For GFAP staining, deparaffinized 5 µm sections were incubated with a polyclonal GFAP antibody (Dako) for one hour. Sections were thoroughly rinsed, and antibody visualized using diaminobenzidine conjugated avidin biotin complex (Vector).

### Lafora body counts

Images from PAS-D slides were acquired at a 400× magnification (Olympus) by a CCD camera (Roper Scientific). Perikaryal and granular (neuronal processes) LB were distinguished by size and location. Numerical density [46] of both perikaryal and granular LB was then determined using the formula:
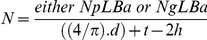
where *N* is the number of either the perikaryal LB or the number of granular LB per unit volume of tissue (number/mm^3^), *NpLBa* is the number of perikaryal LB per area, *NgLBa* is the number of granular LB per area, *d* is the average diameter of either the perikaryal or granular LB, *t* is the thickness of the section (5 µm), and *h* is the smallest recognizable LB (1 µm). A minimum of 500 fields/animal were analyzed using an image analysis program (Image Pro Plus, Media Cybernetics, Bethesda). Data were expressed as means ± SEM and significance calculated using an ANOVA analysis.

### Astrocyte counts

Images from GFAP stained slides were acquired at a 250× magnification using the same microscope and equipment as above. The total number of GFAP positive cells was divided by the total area and expressed as cells/mm^2^. Genotype was blinded to the reviewer. A minimum of 250 fields/animal were analyzed. Images were analyzed using an image analysis program (Image J, NIH, Bethesda). Data were expressed as means ± SEM and significance calculated using ANOVA.

### Glycogen measurements

Mice were sacrificed by cervical dislocation and tissues quickly frozen in liquid nitrogen. Tissues were ground with a mortar and pestle in liquid nitrogen. Aliquots of 30–50 mg of tissue were mixed with 30% potassium hydroxide (KOH) and boiled at 100°C with frequent mixing. Glycogen was then precipitated with a final concentration of 67% ethanol at −20°C, then pelleted. This process was repeated three times. The purified glycogen samples were then dried and suspended in sodium acetate buffer. Glycogen was digested with amyloglucosidase (Sigma) at 37°C. Released glucose was determined using a glucose assay kit (Sigma). The amount of glycogen was calculated and expressed as µmoles of glucose per gram of tissue.

### Electron microscopy

Brains for electron microscopy were taken from mice first perfused through the left ventricle of the heart with 2.5% glutaraldehyde in 0.1 M phosphate buffer (pH 7.4). The tissue was minced into cubic 1 mm blocks and fixed for an additional two to four hours. Samples were then washed in buffer and post fixed in phosphate-buffered 2% OsO_4_ for one hour. They were then dehydrated in an ascending series of acetones prior to being infiltrated, embedded and polymerized at 60°C overnight in embed 812-Araldite. Ultrathin sections were then prepared and stained with uranyl acetate and lead citrate prior to examination and image acquisition in the EM (JEOL JEM 1011, Peabody, MA).

### Myoclonus measurements

Mice were placed in individual Plexiglas chambers and videotaped for four hours. Myoclonus was counted during periods when the animal was not exploring. Myoclonus counts were obtained in periods of a minimum of 10 minutes per mouse. The entire record was reviewed for detection of polymyoclonus. Observer was blinded to genotype. Myoclonus data in Figure 7 is shown as means ± SEM and significance calculated using an unpaired student's t-test.
